# 1871. Lessons Learned from IGRA Test Practices in a Tertiary Hospital

**DOI:** 10.1093/ofid/ofad500.1699

**Published:** 2023-11-27

**Authors:** Farah Daas, Rafael Ruiz-Gaviria, Bhakti Thummar, Naveera Khan, Sheena Ramdeen

**Affiliations:** Medstar Washington Hospital Center, Washington, District of Columbia; Medstar Washington Hospital Center, Washington, District of Columbia; Medstar Washington Hospital Center, Washington, District of Columbia; MedStar Health/Georgetown - Washington Hospital Center Program, Silver Spring, Maryland; MedStar Washington Hospital Center, Washington, District of Columbia

## Abstract

**Background:**

Interferon Gamma Release Assay (IGRA) is frequently ordered in hospitalized patients for latent tuberculosis (TB) screening. Health care workers must be cautioned that acute and critical illness exert a significant effect on IGRA performance, so delaying screening to the outpatient setting is preferred if feasible. When active TB is suspected, IGRA can aid in risk stratification, particularly in areas with low prevalence of TB, but should not be used to confirm or rule out active TB infection. False negative IGRA results in active TB are estimated to be up to 20%. Current guidelines from the Centers for Disease Control and Prevention, the Infectious Diseases Society of America and the American Thoracic Society suggest that IGRA cannot distinguish active from latent TB and recommend prompt microbiological testing when active TB is suspected.

**Methods:**

We conducted a retrospective chart review on hospitalized patients older than 18 years in a tertiary academic center who had an IGRA test done from January 1, 2021, to December 31, 2021. We randomly selected 78 from the 417 identified patients for collection of encounter level data.

**Results:**

IGRA test indications were screening for latent TB in 53.8%, to investigate active pulmonary TB in 30.8%, and for other reasons in 6.4%. IGRA tests had no clear indication in 9% of cases. IGRA test results were 84.6% negative; 10.3% positive; and 5.1% indeterminate. After a negative IGRA, microbiological testing was ordered in 12%, with 1 positive test confirming active TB.

Active TB was suspected in 30.8% of our cohort. Microbiologic testing was done in 41.6% of these patients. In most of the patients with microbiological testing, ordering was delayed until a positive IGRA was available with an average delay of 2.4 days.

**Conclusion:**

Collection of sputum for microbiological testing should not be delayed until IGRA test results are available, and negative results should be scrutinized when suspicion for active TB is high. Caution must be practiced in interpreting IGRA results, and we should not solely rely on IGRA in decision-making regarding isolation, treatment, or further testing. Education of health care staff may be required regarding the indications and timing of IGRA testing.

**Disclosures:**

**All Authors**: No reported disclosures

